# Developmental and age differences in visuomotor adaptation across the lifespan

**DOI:** 10.1007/s00426-022-01784-7

**Published:** 2023-01-09

**Authors:** Marit F. L. Ruitenberg, Vincent Koppelmans, Rachael D. Seidler, Judith Schomaker

**Affiliations:** 1 Department of Health, Medical and Neuropsychology, Faculty of Social and Behavioural Sciences, Leiden University, Pieter de La Court Building, P.O. Box 9555, 2300 RB Leiden, The Netherlands; 2 Leiden Institute for Brain and Cognition, Leiden, The Netherlands; 3 Department of Psychiatry, University of Utah, Salt Lake City, UT USA; 4 Department of Applied Physiology & Kinesiology, University of Florida, Gainesville, FL USA

## Abstract

In the present cross-sectional study, we examined age and sex differences in sensorimotor adaptation. We tested 253 individuals at a local science museum (NEMO Science Museum, Amsterdam). Participants spanned a wide age range (8–70 years old; 54% male), allowing us to examine effects of both development and healthy aging within a single study. Participants performed a visuomotor adaptation task in which they had to adapt manual joystick movements to rotated visual feedback. We assessed the rate of adaptation following the introduction of the visual perturbation (both for early and later stages of adaptation), and the rate of de-adaptation following its removal. Results showed reliable adaptation patterns which did not differ by sex. We observed a quadratic relationship between age and both early adaptation and de-adaptation rates, with younger and older adults exhibiting the fasted adaptation rates. Our findings suggest that both younger and older age are associated with poorer strategic, cognitive processes involved in adaptation. We propose that developmental and age differences in cognitive functions and brain properties may underlie these effects on sensorimotor functioning.

**Supplementary Information:**

The online version contains supplementary material available at 10.1007/s00426-022-01784-7.

## Introduction

Sensorimotor adaptation refers to the adjustment of motor commands and representations in response to changing environmental or internal demands in order to maintain appropriate, goal-directed motor performance. For example, imagine adjusting to the driving characteristics of a rental car on holiday, or to using a mouse that moves the cursor faster than you are used to when using a friend’s computer. The early phase of such adaptation is characterized by fast improvements and is thought to involve strategic, cognitive processes, whereas the late phase is characterized by relatively slow improvements and increasing reliance on automaticity (e.g., Anguera et al., 2010; Eversheim & Bock, 2001; McDougle et al., 2015; Taylor et al., 2014).

The capacity of individuals to modify motor behavior to changing demands (i.e., adaptability) varies across the lifespan. For example, younger children have been found to be less proficient at locomotor adaptation, with children under the age of six years old being unable to adapt completely and slightly older children adapting more slowly compared to adults (e.g., Rossi et al., 2019; Vasudevan et al., 2011). Similarly, younger children have been reported to be poorer at manual adaptation in response to a mechanical force field perturbation (Tahej et al., 2012). In addition, numerous studies have found that older adults show poorer adaptability in comparison to younger adults in both manual adaptation paradigms with visual perturbation (e.g., Anguera et al., 2011; Bock, 2005; Fernandez-Ruiz et al., 2000; Seidler, 2006, 2007; Wolpe et al., 2020) and mechanical perturbation (e.g., Huang & Ahmed, 2014). In contrast, other studies using locomotor adaptation paradigms did not observe differences in adaptation rates between younger and older adults (e.g., Bakkum et al., 2021; Malone & Bastian, 2016; Vervoort et al., 2019; but see Fettrow et al., 2021). Taken together, prior findings hint at an inverted-u relationship between age and sensorimotor adaptability across the lifespan with performance peaking in young adulthood. Moreover, prior work suggests that, in particular, the strategic, explicit components of adaptation are sensitive to age differences, whereas implicit, more automatic mechanisms may be relatively unaffected. Specifically, studies on healthy aging provide indications that reduction in motor adaptability with older age is driven by a decline in explicit and working memory systems (Anguera et al., 2011; Vandevoorde & Orban de Xivry, 2019; Wolpe et al., 2020), and that in children implicit sensorimotor adaptation matures earlier than explicit spatial representation (Tahej et al., 2012). However, the hypothesis that explicit components are particularly sensitive to age differences has not systematically been tested within a single sample across the lifespan, as previous studies had either a developmental or healthy aging approach focusing on group differences relative to young adults. Moreover, prior studies have evaluated smaller samples (typically up to 30 participants per age group), potentially contributing to some of the mixed evidence described above.

In addition to the effects of age, there may also be effects of sex on sensorimotor adaptation as men and women differ in many sensory systems, neural anatomy, and functional responses (Mark et al., 2014). Indeed, one study by Moreno-Briseño et al. (2010) has hinted at sex-differences in adaptability. They examined how men and women adapted their ball throwing performance to a 30 degree visual distortion induced via prism lenses. While they did not observe adaptation differences between groups, they found that women showed significantly larger aftereffects which could suggest that sensorimotor representations were updated more strongly in women than in men. The authors proposed that women relied more on implicit, more automatic spatial alignment processes that reorganize visual and motor information, which led to larger aftereffects. However, this was not supported in a study by Wolpe et al. (2020), who controlled for sex in their statistical model to test the effects of healthy aging on manual adaptation. While they found the aforementioned effect of age, they did not observe effects of sex. Overall, reports on sex differences in sensorimotor adaptation thus are scarce and findings are inconsistent.

In the present study, we investigated age and sex differences in sensorimotor adaptation across the lifespan. As part of a public science experiment, we had participants spanning a wide age range (8–70 years) complete a manual adaptation task. We hypothesized that the relationship between age and adaptability would be quadratic, with both younger and older individuals showing poorer adaptation compared to individuals more towards the middle of our age range. In particular, this was expected for the relatively early, explicit phase of adaptation. Using an explorative approach, we also evaluated whether there were sex differences in adaptability.

## Methods

### Participants

The sample included 253 participants aged 8 to 70 years (median age = 17 years, interquartile range 10–39 years; 54% male). According to self-report data, 229 participants were right-handed, 19 were left-handed, and 5 were ambidextrous. Participants were part of a larger study that examined novelty effects on learning and was conducted at *Science Live*, an innovative research program of the NEMO Science Museum in Amsterdam, the Netherlands which gives visitors the opportunity to volunteer to participate in scientific research. Results regarding the novelty manipulation have been reported elsewhere (Ruitenberg et al., 2022; Schomaker et al., 2022); here, we present novel findings and only analyze data from participants in the control group (i.e., 253 individuals not subjected to the novelty manipulation). Written informed consent was obtained from all participants or their parents in the case of minors. The study was approved by the Psychology Research Ethics Committee of Leiden University.

### Motor adaptation task

**Fig. 1:**
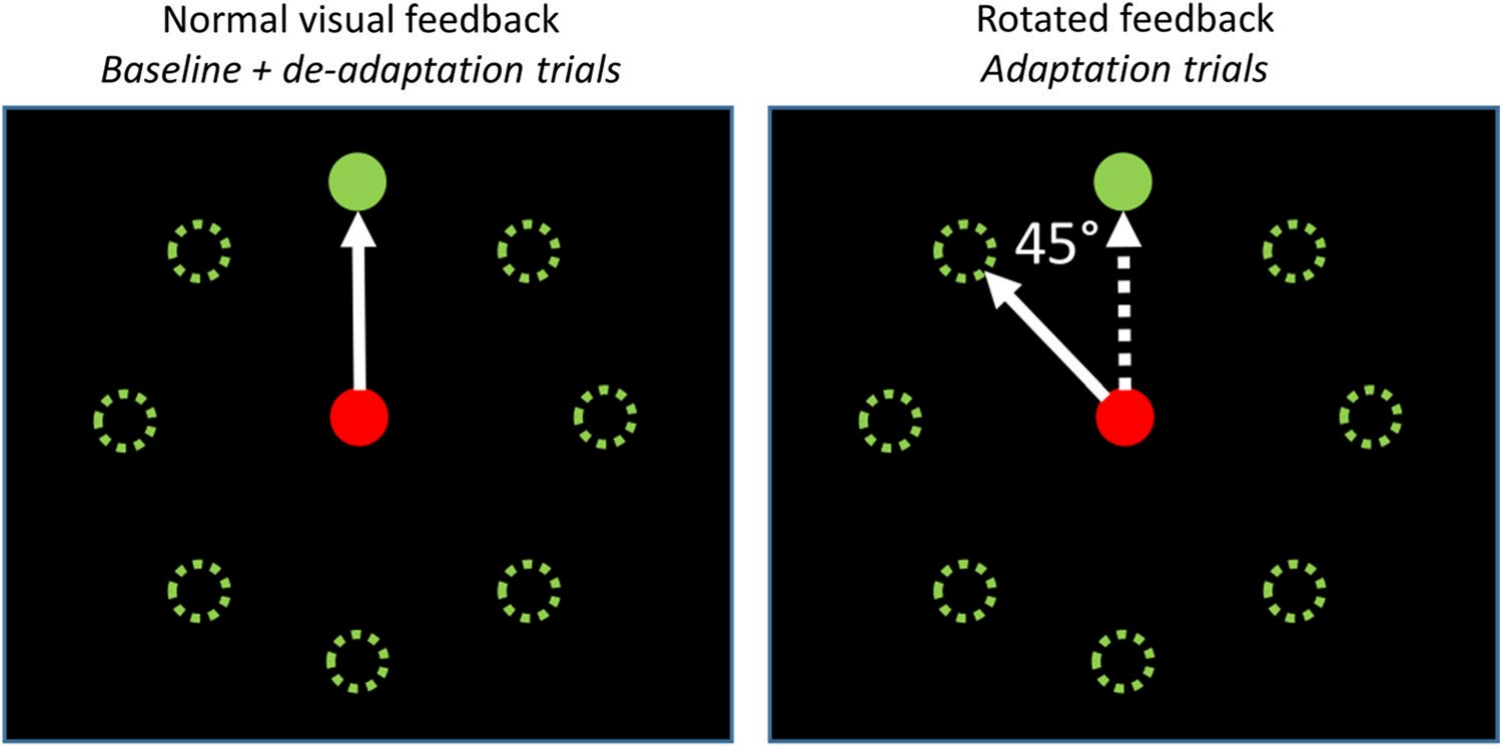
Illustration of stimulus and feedback presentation in our visuomotor task. During baseline and de-adaptation trials, the movements of the red cursor corresponded to path of the joystick movement. During adaptation trials, the movement of the cursor was rotated by 45° counterclockwise relative to the joystick movement

The manual visuomotor adaptation task used in the present study has been used extensively to study adaptation learning by us and others (e.g., Anguera et al., 2010, 2011; Lametti et al., 2020; Mazzoni & Krakauer, 2006; Ruitenberg et al., 2018a, 2018b; Sainburg & Wang, 2002; Seidler, 2005, 2006; Seidler et al., 2006). A detailed description of the current version of the task is provided in Ruitenberg et al. (2022). In brief, participants used a dual axis joystick with their preferred hand to hit targets presented on a laptop screen. The joystick controlled a red circle (i.e., the cursor) that was presented at the central position on the screen when the joystick was also centered. At the start of each trial, a green target circle was presented for 1000 ms at one of eight equidistant locations 4.6 cm from the center of the screen (Fig. 1). Participants were instructed to move the red circle onto the green circle as quickly as possible by moving the joystick, and to relax their force on the joystick handle after target disappearance to allow the cursor to recenter for the next trial. Each movement was initiated from the central position on the screen and the target circle randomly appeared in one of the eight possible locations once in every eight trials; each repetition of eight trials was considered a block. Participants first completed one block of practice trials under normal visual feedback. They then started the task by performing eight trials under normal visual feedback (i.e., baseline trials; one block) followed by 40 trials under 45° counterclockwise rotated feedback (i.e., adaptation trials; five blocks). Finally, participants completed another 16 trials under normal visual feedback, which allowed us to measure the aftereffects of adaptation (i.e., de-adaptation trials; 2 blocks). Stimulus presentation, timing, and data recording were controlled by PsychoPy software (version 1.84.1, running under Windows 10), and movements were made using a self-centering joystick (Logitech G Extreme 3D Pro) connected to a Dell Latitude 5580 laptop.

Motor performance was assessed by measuring direction error, defined as the angle between the straight line from the start position to the target and the line from the start position to the cursor’s position at the time of peak movement velocity (cf. Anguera et al., 2010, 2011; Lametti et al., 2020; Seidler, 2005, 2006; Seidler et al., 2006). Depending on the participants’ joystick movement during a trial relative to the target location, these direction errors could be both positive and negative. As participants would occasionally attempt to guess where the target would appear and move the joystick in a (wrong) direction of their choice without attending to the location of the target, trials in which the direction error deviated more than 2.5 standard deviations from the mean were replaced by the mean subject-specific direction error across all trials of a phase (i.e., baseline, adaptation, de-adaptation), to minimize the influence of such unrepresentative guessing-trials. This resulted in the replacement of 2.2% of the trials overall. We then determined the adaptation rate to operationalize adaptability within our current experimental task, by calculating the decay constant across adaptation trials (fit using an exponential decay regression function, where *x* is the trial number and *y* is the natural log of the direction error) and used this score as the primary outcome measure for studying adaptation (cf. Morehead et al., 2015; Ruitenberg et al., 2018b; Zarahn et al., 2008). This was done for all 40 adaptation trials from each subject to obtain an estimate of the adaptation rate constant, as well as separately for the first and second half (i.e., 20 trials each) of all adaptation trials to distinguish between relatively early and late adaptation. Finally, we also determined the adaptation rates across all 16 de-adaptation trials.

### Procedure

All participants provided written informed consent; in case the participant was a minor, their parent or legal guardian was asked to complete a consent form on their behalf. As part of the larger study, participants performed a battery involving different tasks including the exploration of two virtual environments. Participants in the present study all explored the same virtual environment twice and were not exposed to novelty; they thus served as control subjects for the overall study (Ruitenberg et al., 2022; Schomaker et al., 2022). They subsequently performed a word-learning task, the motor adaptation task, and a landmark memory task (for details and results regarding the word learning and landmark tasks, see Schomaker et al., 2022). The entire experimental procedure took about 20–25 min per participant, and the visuomotor adaptation task approximately 3 min.

## Results

### General motor adaptation patterns

**Fig. 2:**
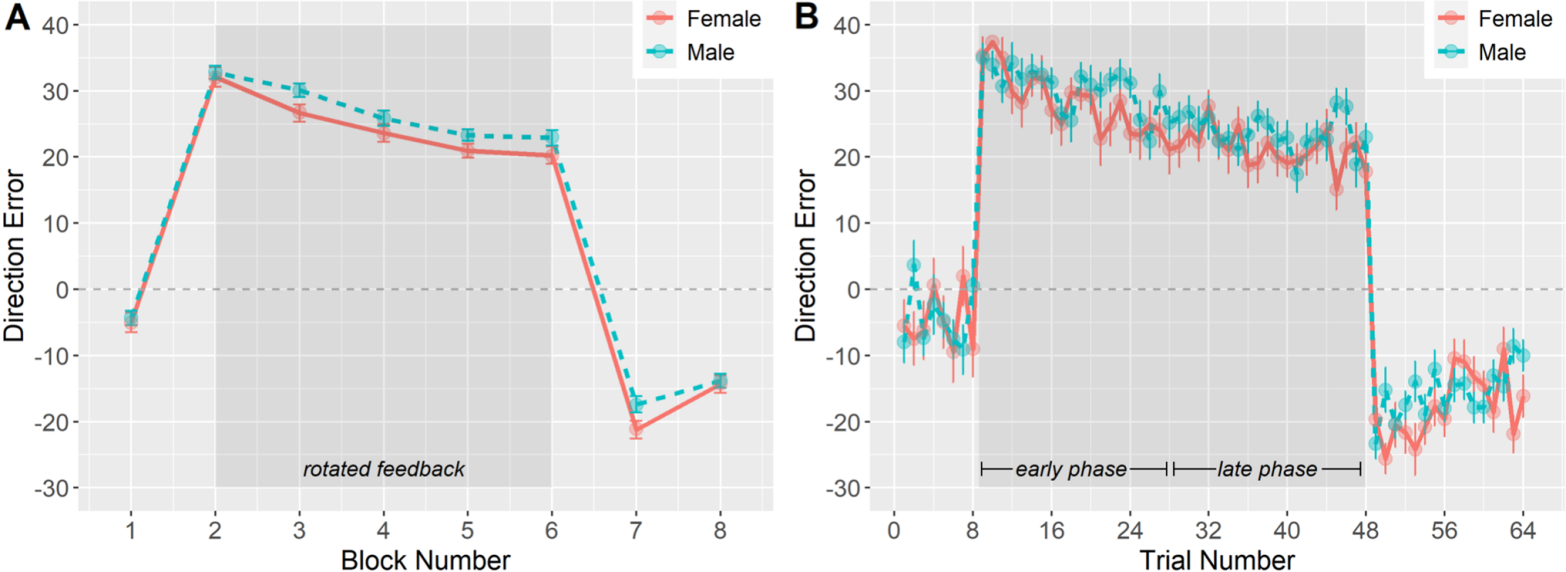
Mean direction error per block (**A**; 1 = baseline, 2–6 = adaptation, and 7–8 = de-adaptation) and across all trials (**B**) as a function of sex in the manual adaptation task. Shaded areas represent blocks and trials with rotated feedback. Error bars represent standard errors

To examine whether our data were in line with the aforementioned general pattern observed in sensorimotor adaptation studies, we first performed a mixed ANCOVA on direction error with sex (2; male vs. female) as a between-subject variable, block (8; blocks 1–8) as a within-subjects variable, and age as a covariate. Results showed an effect of block, *F*(7,1743) = 210.89, *p* < 0.001, *η*_*p*_^2^ = 0.46. Figure 2A shows that, in line with the typical adaptation pattern, participants’ direction error increased when the rotated feedback was introduced in the first adaptation block but then gradually improved during the subsequent adaptation blocks. When the rotation was removed for the de-adaptation blocks, participants had to readapt to the veridical feedback, leading to initial overshooting of the target in the opposite direction of that induced by the perturbation. Example single subject data patterns are presented in Supplementary Figure S1.

Results further showed that across all experimental blocks direction errors were larger in males than females (12.42 vs. 10.38), *F*(1,249) = 7.11, *p* = 0.008, *η*_*p*_^2^ = 0.03. We also observed a main effect of age, *F*(1,249) = 4.01, *p* = 0.046, *η*_*p*_^2^ = 0.02, and an interaction effect between block and age, *F*(7,1743) = 2.74, *p* = 0.008, *η*_*p*_^2^ = 0.01. Post hoc tests per block indicated that age was significantly related to average direction error in B2, B4, and B5, *Fs*(1,249) > 5.32, *ps* < 0.022, but not the other blocks (*ps* > 0.20), with older age being related to larger direction errors. There was no significant block × sex interaction or three-way interaction (*ps* > 0.35). For illustration purposes, we also present the trial-by-trial performance data across participants in Fig. 2B.

### Adaptation rates

We performed an ANCOVA on overall adaptation rates with sex as a between-subject variable and age as a covariate to examine the effects of sex and age on adaptability. Results showed an effect of age on adaptation rate, *F*(1,249) = 11.83, *p* < 0.001, *η*_*p*_^2^ = 0.05. When further examining this effect via a planned quadratic^1^ regression analysis including age as predictor and adaptation rates as the outcome measure, we found that both younger and older age were associated with slower adaptation, *F*(2,250) = 27.26, *p* < 0.001, *R*^*2*^ = 0.18. Adaptation rates did not significantly differ between male and female participants (− 0.012 ± 0.011 vs. − 0.014 ± 0.011; *p* = 0.28), and there was no significant age × sex interaction, (*p* = 0.54).

**Fig. 3:**
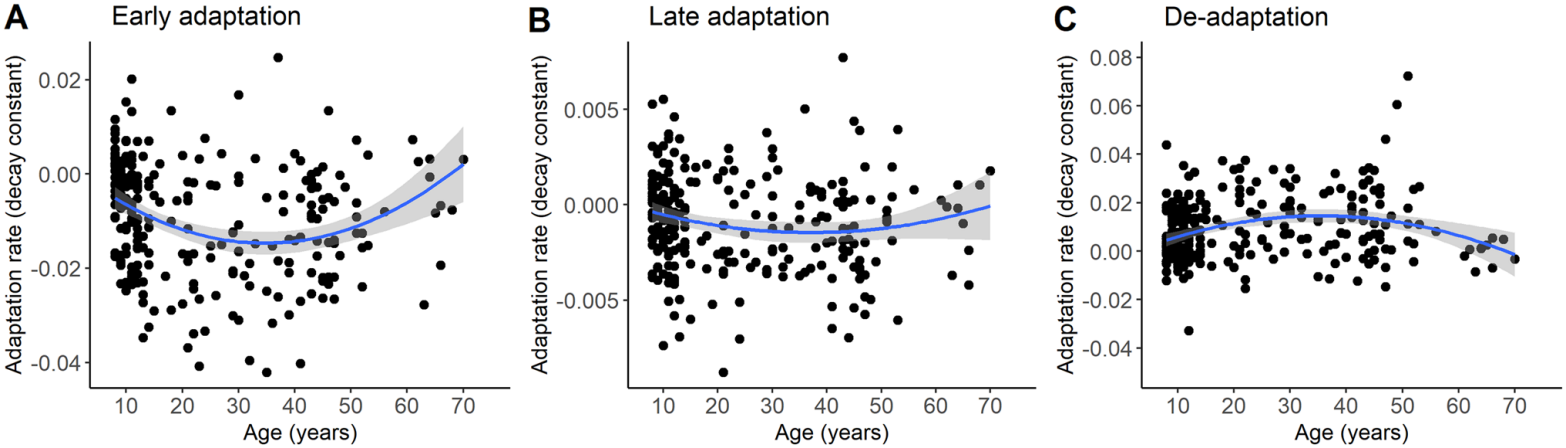
Scatter plots showing the quadratic association between age and adaptation rates across early adaptation trials (**A**), late adaptation trials (**B**), and de-adaptation trials (**C**). More negative adaptation rates and more positive de-adaptation rates indicate a steeper decay over the trials (i.e., faster improvement). Note that the y-axes have different scales; shaded areas denote the 95% CI

To evaluate differences in adaptation in the relatively early and later phases of the task, we ran separate analyses on adaptation rates that were calculated across the first half of adaptation trials and those calculated across the second half of adaptation trials. For the early phase, results showed an effect of age on adaptation rate, *F*(1,249) = 4.78, *p* = 0.030, *η*_*p*_^2^ = 0.019. When further examining this effect via a post-hoc quadratic regression analysis, we found that both younger and older age were associated with slower early adaptation, *F*(2,250) = 13.30, *p* < 0.001, *R*^*2*^ = 0.09 (Fig. 3A). Early adaptation rates did not significantly differ between male and female participants (− 0.009 ± 0.012 vs. − 0.012 ± 0.012; *p* = 0.31), and there was no significant age × sex interaction, (*p* = 0.76). For the late phase, results showed no significant main or interaction effects of age and sex (*ps* > 0.10; Fig. 3B), suggesting that adaptation during this phase was similar across participants. We also directly compared adaptation rates between the early and later phases of the task, to substantiate our assumption that these relate to different processes. Results of a paired *t* test confirmed that adaptation rates were steeper (i.e., decay constants were more negative) for the early than the late phase (− 0.009 ± 0.012 vs. − 0.001 ± 0.003), *t*(252) =  − 11.76, *p* < 0.001.

Next, we ran an ANCOVA on adaptation rates across de-adaptation trials. Again, results showed a significant effect of age, *F*(1,249) = 5.13, *p* = 0.024, *η*_*p*_^2^ = 0.02. Results of a post-hoc quadratic regression analysis showed that both younger and older age were associated with slower de-adaptation, *F*(2,250) = 11.10, *p* < 0.001, *R*^*2*^ = 0.08 (Fig. 3C). Similar to the adaptation phase, de-adaptation rates did not significantly differ between male and female participants (0.009 ± 0.013 vs. 0.010 ± 0.014; *p* = 0.64), and there was no significant interaction effect (*p* = 0.78).

Finally, we checked whether the pattern of results remained stable when adjusting for individual differences in initial bias to the rotated feedback. That is, we examined whether the error during the first block of adaptation was related to overall adaptation rate. To that end, we reran our ANCOVAs on adaptation rates while including the mean direction error from the first adaptation block as an additional covariate. Results confirmed that even when correcting for initial biases, the effect of age remained significant for the overall adaptation rate, *F*(1,248) = 11.75, *p* < 0.001, *η*_*p*_^2^ = 0.045, early adaptation rate, *F*(1,248) = 4.76, *p* = 0.030, *η*_*p*_^2^ = 0.019, and de-adaptation rate *F*(1,248) = 5.11, *p* = 0.025, *η*_*p*_^2^ = 0.020.

### Onset of developmental and aging differences

To further examine the age of onset for the developmental and aging differences in adaptability in the early and de-adaptation phases, we first identified the age at which adaptation rates were estimated to be optimal in the quadratic models; for both the early and de-adaptation phases, this was at 35 years of age. We then used locally weighted polynomial regression (LOESS) smoothing to describe the age trajectories (see Westlye et al., 2010; Chan et al., 2014). This approach is even more robust to variations in the age range than quadratic regression (Fjell et al., 2010) and allows us to determine the age at which the adaptation rates started to develop and deteriorate by identifying the inflection point for each of these phases. Results revealed that adaptation rates started to improve at 24 years for the early adaptation phase and at 20 years for the de-adaptation phase. Furthermore, results showed that these rates declined at 46 and 49 years of age for the early and de-adaptation phases, respectively.

## Discussion

The current study is the first to investigate the relationship between age and sensorimotor adaptation within a single, lifespan sample. We observed a typical adaptation pattern across our group of participants aged 8 to 70 years, supporting the notion that our experimental paradigm translates well from a controlled laboratory setting to the relatively uncontrolled setting of a science museum. Our results further revealed a quadratic relationship between age and adaptability, with both younger and older participants showing slower adaptation and de-adaptation rates than those in the middle of the age range. More specifically, we observed an inverted *u*-shaped relationship between age and early adaptation rates, suggesting that age affects the relatively early strategic, cognitive processes. We did not see this effect for adaptation rate calculated across the second half of our adaptation trials. This suggests that age does not impact the later, more implicit processes of adaptation; however, given the relatively small number of trials that participants performed, it could be argued that the current task may not be suited to adequately assess this and we acknowledge that replication therefore is necessary. Finally, we found no indications that sex affected visuomotor adaptation rates, although there was an overall smaller error for females than males across all study blocks.

Several studies comparing younger and older adults propose that age-related reductions in sensorimotor adaptation may be linked to an age-related reduction in cognitive functions involved in such adaptation (e.g., Li et al., 2021; Vandevoorde & Orban de Xivry, 2019). For example, earlier work has demonstrated that spatial working memory—which could be used to apply an explicit aiming strategy—was positively associated with adaptability (Anguera et al., 2010, 2011; Ruitenberg et al., 2018a). In addition, inhibitory control has been shown to be positively associated with adaptability (Li et al., 2021; Simon & Bock, 2017). Such control may be necessary to suppress typical, unadapted movement plans that are not functional for dealing with the perturbation. As these functions are known to not just decline with older age but to also still be in development during childhood and adolescence (Conklin et al., 2007; Isbell et al., 2015), this could explain our observed quadratic relationship between age and adaptability.

Neuroimaging studies also support the notion that sensorimotor adaptation involves a combination of more cognitive and procedural processes that are differentially affected by age. For example, Anguera et al., (2010, 2011) found that both early rates of visuomotor adaptability and spatial working memory were linked to activation in the dorsolateral prefrontal cortex for younger but not older adults. More recently, Wolpe et al. (2020) demonstrated that age differences in adaptation within an adult cohort aged 18–89 years were related to smaller gray matter volume in the striatum and prefrontal cortex (thought to be involved in explicit processes of adaptation), but not in the cerebellum (thought to be involved in implicit processes). Similar to the cognitive functions described above, these brain areas are known not just to be affected by aging but to also be slow to develop, particularly prefrontal cortex (up to 20–25 years old; Amso & Casey, 2006). Our observation that adaptation rates started to improve in the early 20s fits this trajectory. We further observed that declines in adaptability start to emerge around the mid-40s. This is in line with reports that in particular explicit components of adaptation are already affected at pre-retirement adult age (Heuer et al., 2011). A similar age of onset has been described for other domains, including sensorimotor inhibitory function (Ruitenberg et al., 2019), bimanual coordination (Boisgontier et al., 2018), and cognitive function (Singh-Manoux et al., 2012). Our findings, in combination with the behavioral and neuroimaging literature, thus support a shared cognitive resource hypothesis from both a developmental and aging perspective to explain the quadratic relationship between age-related differences across the lifespan in explicit, strategic processes involved in visuomotor adaptation and cognitive functions.

Notable strengths of the present study are its lifespan approach and sample size. Whereas the majority of previous studies examining age effects on sensorimotor adaptation compared performance of young adults to that of either children or older adults, our study included both a developmental and healthy aging perspective in which we used age as a continuous variable. Moreover, our sample size greatly exceeds those reported in prior investigations on group-based age or sex differences in sensorimotor adaptation (i.e., typically *n* = 10–30 per group; with exception of Wolpe et al., 2020, who included *n* = 322 adults and conducted analyses using age as a continuous variable). A limitation of our study is that the designation of the early and late adaptation phases are made rather arbitrarily within the field of sensorimotor adaptation. That is, most studies on sensorimotor adaptation define the early and late phase within the context of their design, depending for example on the duration of the experiment and the total number of adaptation trials. Possibly the current design may have obscured relatively smaller sex differences between early and late adaptation that arise even later during learning. Moreover, we acknowledge that our visuomotor task does not formally distinguish between measures of implicit and explicit contributions to adaptation. As such, future studies on lifespan differences in sensorimotor adaptation should consider employing dedicated tasks (e.g., an error clamp paradigm; Morehead et al., 2017) to obtain distinct measures for these processes. This may also help to elucidate the relationship and joint contribution of these processes in adaptation (Maresch et al., 2021) and the extent to which individuals may be able to compensate for specific declines in one process by relying more on the other. Furthermore, as our experimental procedure only included one type of sensorimotor adaptation, namely adaptation of hand movements to rotated visual feedback, the generalizability of findings to other types of adaptation is unclear. Future studies should examine lifespan differences in manual adaptation to other types of perturbations (e.g., force-field reaching; prism adaptation) and in other modalities of adaptation (e.g., locomotor adaptation, which involves bilateral whole-body control), as these may recruit different cognitive processes and/or brain areas and thus be differently affected by age and/or sex. Finally, future studies could investigate potential age and sex differences in retention and savings of adaptation. Participants in the present study performed the manual adaptation task only once, but previous studies have shown that participants adapt faster when they are re-exposed to the same perturbation thus suggesting that changes in sensorimotor representations and/or memories of adaptation strategies persist after the initial training session. Such savings of adaptation have been observed 1 day after initial performance (Seidler et al., 2017), several months later (Ruitenberg et al., 2018b), and even 1 year after initial adaptation (Landi et al., 2011).

Overall, our findings demonstrate an inverted *u*-shaped relationship between age and sensorimotor adaptation across the lifespan. Both younger and older participants specifically show slower adaptation in the relatively early stages that are thought to rely on strategic, cognitive processes, but not later stages that are thought to involve implicit processes. Our findings underline the importance of accounting for age differences in visuomotor adaptation research when for example comparing healthy and clinical groups. In addition, these results could have implications for the design of neuropsychological rehabilitation programs in which patients with congenital or acquired brain damage must (re)learn sensorimotor skills. For instance, programs may be tailored for patients from certain age groups by offering more frequent or longer training sessions that tap into implicit learning processes for younger and older individuals. In addition, it may allow for the development of interventions that can potentially counteract and/or slow age-related declines in motor (re)learning.

## Supplementary Information

Below is the link to the electronic supplementary material.

Supplementary file1 (TIFF 701 KB)

## Data Availability

The data that support the findings of this study are available on request from the corresponding author.

## Footnotes

1 We also ran linear regression analyses for all age effects; AIC comparisons showed that the quadratic models consistently had a better fit than the linear models (i.e., ∆ > 2).
