# Supplementary figures and images for: Developmental and age differences in visuomotor adaptation across the lifespan
Source: Psychol Res. 2023 Jan 9;87(6):1710–7. doi: 10.1007/s00426-022-01784-7 (PMC10366290; doi:10.1007/s00426-022-01784-7)

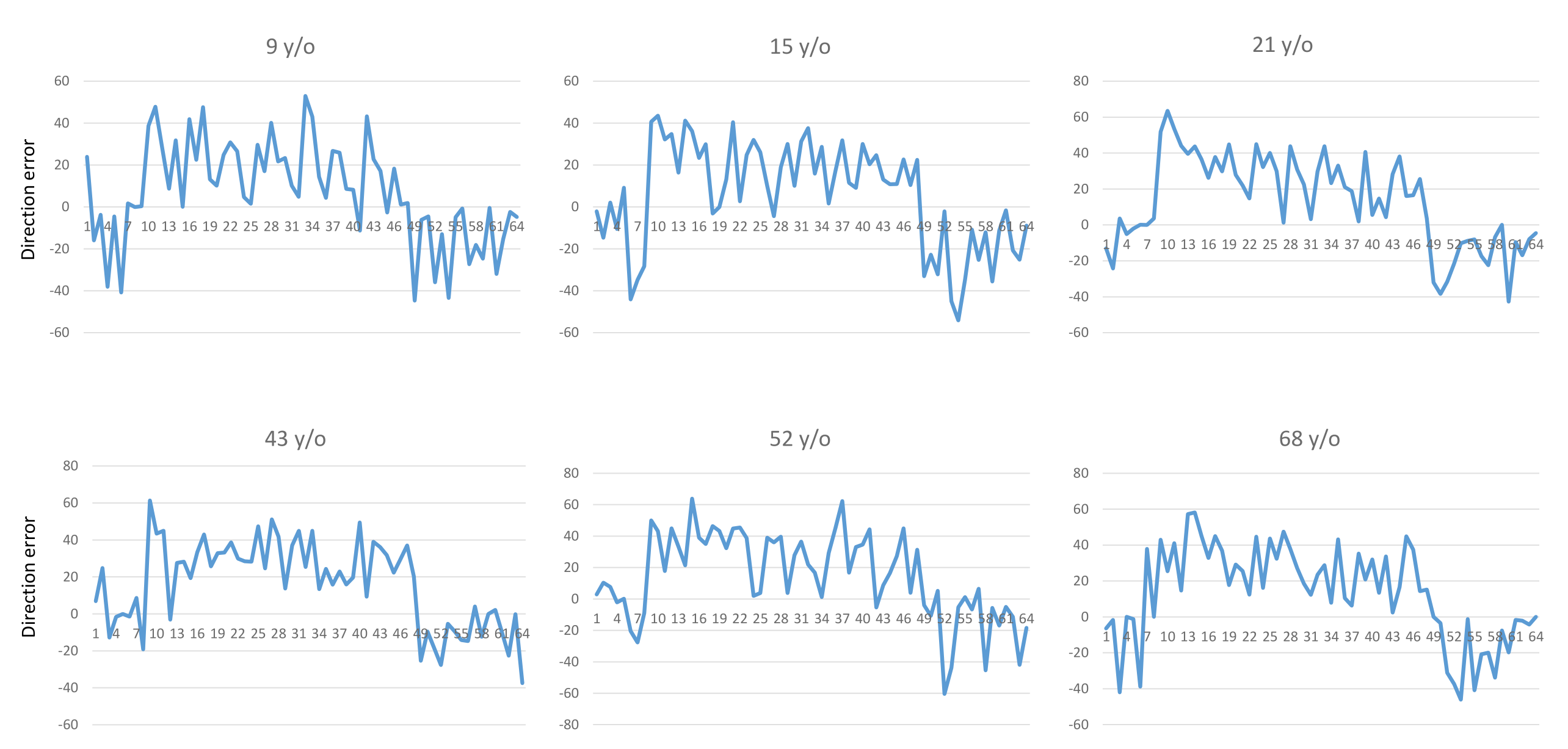

Supplement: Supplementary file 1 — Supplementary file1 (TIFF 701 KB) [file 426_2022_1784_MOESM1_ESM.tiff]
